# Enhancing pooling levels strengthens the risk resilience of healthcare insurance: a case study of basic medical insurance fund operations data in Gansu, China

**DOI:** 10.1186/s12889-024-18558-y

**Published:** 2024-04-23

**Authors:** Feng Hu, Liu Heming, Cao Wenxuan, Wang Xuemei, Liang Qijun, Hu Xiaobin

**Affiliations:** 1 Institute of Epidemiology and Health Statistics, School of Public Health, Lanzhou University, 730000 Lanzhou, Gansu China; 2 Gansu Provincial Medical Insurance Service Centre, 730000 Gansu, China

**Keywords:** Basic Medical Insurance, Enhancing pooling levels, DEA model, COVID-19

## Abstract

**Background:**

In China, enhancing the pooling levels of basic health insurance has consistently been regarded as a pivotal measure to promote the refinement of the healthcare insurance system. From 2020 to 2022, the widespread outbreak of COVID-19 posed new challenges to China’s basic health insurance.

**Methods:**

The research utilizes Data Envelopment Analysis (DEA), Malmquist index assessment, and fixed-effects panel Tobit models to analyze panel data from 2020 to 2022, assessing the efficiency of basic health insurance in Gansu Province.

**Results:**

From 2020 to 2022, the average overall efficiency of the municipal pooling of Basic Medical Insurance for Urban and Rural Residents was 0.941, demonstrating a stable trend with a modest increase. The efficiency frontier regions have expanded from 5 (35.71%) to 7 (50%). Operational efficiency exhibited a negative correlation with per capita hospitalization expenses and per capita fund balance but a positive correlation with per capita accumulated fund balance and reimbursement rates for hospitalized patients. In 2021, compared to 2020, the county-pooling Basic Medical Insurance for Urban Employees saw a decline of 0.126 in overall efficiency, reducing the efficiency frontier regions from 8 to 3. However, from 2021 to 2022, the municipal-coordinated Basic Medical Insurance for Urban Employees experienced a 0.069 increase in overall efficiency, with the efficiency frontier regions expanding from 3 to 5. Throughout 2020 to 2022, the operational efficiency of the Urban Employee Basic Medical Insurance showed a consistent negative correlation with per capita fund balance.

**Conclusion:**

From 2020 to 2022, the overall operational performance of basic health insurance in Gansu Province was satisfactory, and enhancing the pooling level is beneficial in addressing the impact of unforeseen events on the health insurance system.

## Introduction

### Background

Health holds paramount importance on a global scale [1]. The inception of a robust medical insurance system holds immense significance in preserving public health, promoting health awareness, optimizing the allocation of medical resources, and sustaining social progress [2]. The basic medical insurance system reflects the crucial emphasis of the state and society on the health and welfare of the populace. It is essential for promoting the overall health of the population and public health [3, 4]. China’s social medical insurance system has undergone significant evolution since the establishment of urban employees’ basic medical insurance (UEBMI) in 1998 and the subsequent introduction of the new rural cooperative medical care system in 2003 [5]. The completion of nationwide coverage occurred in 2009 with the establishment of urban resident basic medical insurance [6]. In 2016, China’s State Council issued opinions on integrating urban and rural basic medical insurance systems, merging urban residents’ basic medical insurance and new rural cooperative medical care into a unified system to enhance operational efficiency.

However, in the ongoing process of enhancing basic medical insurance, the challenge of the pooling level, commonly referred to as “vertical fragmentation,” persists and requires further attention [7, 8]. The term “vertical fragmentation” within the healthcare insurance system denotes the presence of independent policies and operational regulations across various administrative levels, including provinces, cities, and counties. This results in a fragmented state within the entire healthcare insurance system. In China’s basic medical insurance system, this phenomenon is notably conspicuous, primarily manifesting through institutional fragmentation, unequal benefits, and a lack of coordination in reform initiatives. Consequently, basic medical insurance at lower pooling levels is suggested to have a constrained ability to manage risks during unforeseen public events [6, 9]. Upgrading the level of pooling is considered to be the main means of increasing the operational efficiency of health insurance and improving its risk resistance.

Municipal pooling of basic medical insurance has been extensively implemented across China [10]. In response to the challenge of vertical fragmentation, Gansu Province, in 2019, elevating the pooling of URRBMI to the municipal level, guided by the Gansu Province Municipal Pooling of Urban and Rural Residents’ Basic Health Insurance Implementation Opinions policy. And, in 2021, the Gansu Provincial Health Insurance Bureau and the Department of Finance issued a Circular on Further Implementing Municipal Pooling of Urban Employees’ Basic Medical Insurance. This circular stipulated that all municipalities in Gansu Province were to achieve municipal pooling of UEBMI by January 1, 2022.

### Literature review

Elevating the standard of fundamental health insurance pooling is widely recognized as pivotal for addressing “vertical fragmentation,” enhancing operational efficiency within the health insurance system, and bolstering its risk tolerance. Smith P. C.‘s examination of diverse risk pooling models highlights the detrimental effects of coordination deficiencies on the health system, advocating for heightened pooling levels [11]. Similarly, Ali Ahangar argues for increased pooling levels in health insurance, citing the scale and uncertainty of individual medical expenditures, which, when addressed, can reduce uncertainty and facilitate effective risk-sharing [12]. Naoki Ikegami and peers qualitatively analyze Japan’s universal health insurance evolution, underscoring the necessity of elevated pooling levels for long-term sustainability and equity [13]. Shibuya et al. identify threats to Japan’s universal health insurance sustainability and propose elevating pooling levels as a safeguard [14]. Additionally, McIntyre et al.‘s retrospective study on South Africa’s health insurance development emphasizes the importance of establishing an integrated fund pool to rectify resource disparities between public and private sectors [15].

Research on enhancing the pooling level of basic medical insurance in China encompasses empirical, theoretical, necessity, and factor analysis studies. Luo Jiaying construct a provincial-level model for UEBMI in Fujian Province, utilizing hierarchical analysis with data on insurance participation across coordination areas, advocating for its feasibility [16]. Fu Mingwei. address the transition from municipal to provincial-level pooling, employing a Probit model for empirical analysis, marking a significant empirical inquiry into provincial pooling [17]. Some scholars offer qualitative insights on elevating China’s basic medical insurance pooling level [18, 19]. Regarding influencing factors, Li Yaqing identifies moral hazard escalation as a risk factor post-coordination level increase [20]. Fu Mingwei, utilizing a Probit model, identify urban workers’ insurance participation and the proportion of financially governed counties and districts as pivotal factors influencing provincial-level coordination advancement [17]. Qingyue Meng, synthesizing domestic and international studies on insurance integration, assert its significance in reducing inter-scheme disparities, enhancing risk-sharing, service coverage, financial protection, operational efficiency, and health system integration [6]. They stress the broader service and protection for rural residents, the elderly, and the chronically ill, along with addressing challenges posed by population mobility [6].

In the realm of health insurance pooling elevation, research has predominantly focused on delineating its benefits, imperative, strategies, and influencing factors. However, extant studies regarding how augmenting basic health insurance levels can bolster risk resilience primarily lean on the “law of large numbers” as a theoretical foundation. Hence, this study delves into pertinent research on advancing health insurance coordination through the following avenues: (i) Utilizing operational data from Gansu Province’s basic health insurance fund spanning 2020–2022, we employ the DEA model to assess the efficiency of UEBMI alongside URRBMI. (ii) Considering the disparate municipal pooling attainment timelines for the two basic health insurance types in Gansu Province, China, we analyze the COVID-19 pandemic’s impact on system operational efficiency across varied pooling levels, comparing the URRBMI with the UEBMI and assessing enhancement pooling efficiency scores. (iii) Employing the Malmquist index and panel data Tobit model, we comprehensively scrutinize basic health insurance operational efficiency across different periods, probing its influential factors. Ultimately, our study furnishes empirical evidence supporting the notion that elevating basic medical insurance pooling levels can enhance fund operational efficiency and fortify its resilience against risks, thereby furnishing a groundwork for policy formulation geared toward pooling enhancement in medical insurance.

**Fig. 1:**
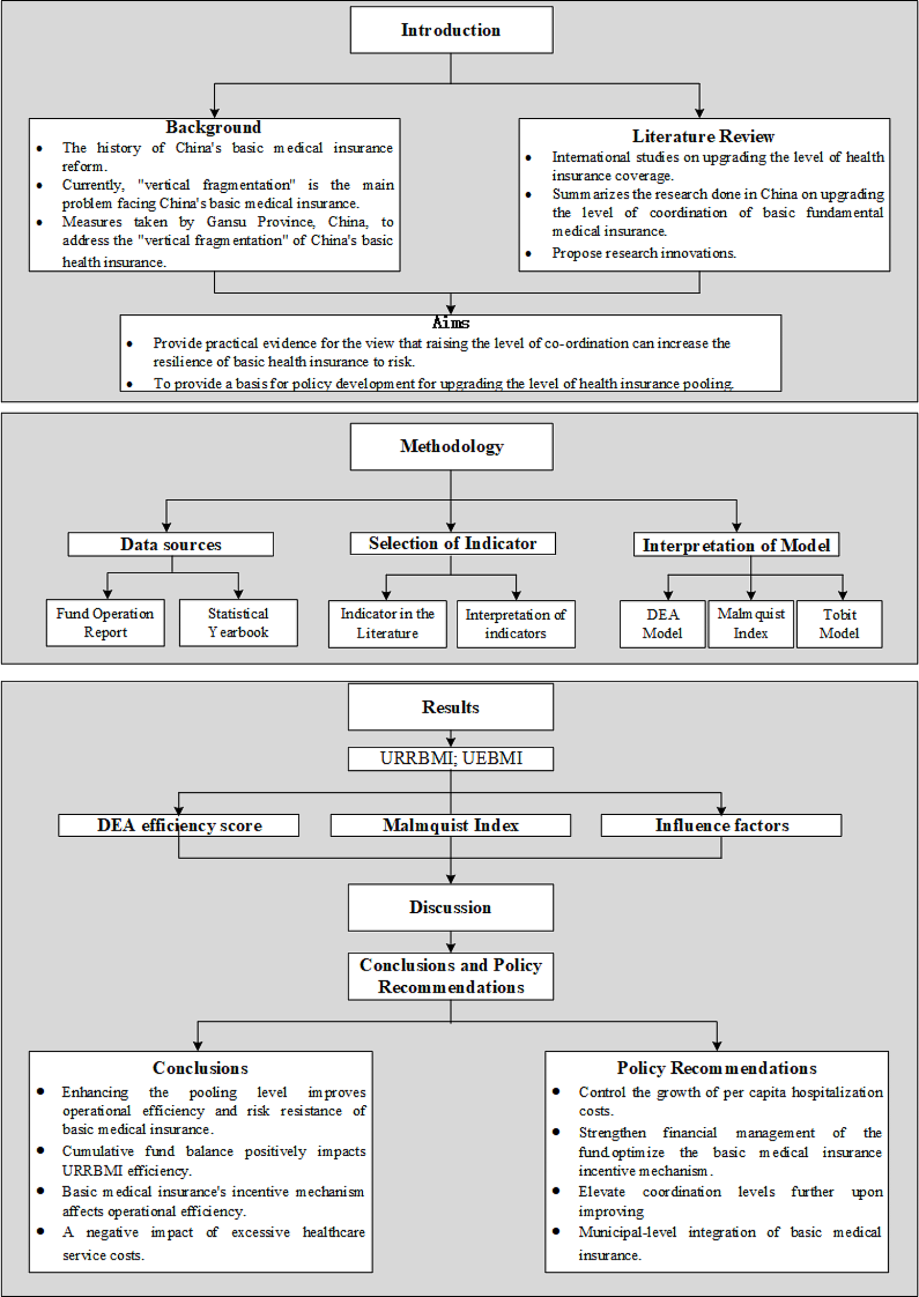
Research framework

To facilitate reader comprehension, a graphical representation illustrating the research framework has been devised (Fig. 1).

## Methodology

### Data sources

The data employed in this study comprises 14 variables, covering 14 cities in Gansu Province from 2020 to 2022. These variables constitute panel data. Specifically, the Fund Operation Report of the Gansu Provincial Bureau of Medical Security provided data on fund income (in RMB 100 million), fund expenditure (in RMB 100 million), the number of insured individuals, fund balance (in RMB 100 million), cumulative fund balance (in RMB 100 million), hospitalization rate (%), and the actual reimbursement ratio of hospitalized patients (%) for both URRBMI and UEBMI. Furthermore, data on the number of health technicians per 10,000 people for the years 2020–2021, the average salary of the urban population, GDP per capita, and year-end population figures were obtained from the Gansu Provincial Statistical Yearbook (2021–2022) [21]. For 2022, data from the Gansu Provincial Bureau of Statistics Statistical Bulletin of National Economic and Social Development of Cities and Prefectures (2022) [22]. The Gansu Provincial Health and Wellness Statistical Yearbook (2021–2023) provided data on per capita outpatient expenses (in RMB), per capita inpatient expenses (in RMB), the number of medical institutions, and the number of tertiary hospitals.

### Selection of indicators

Selection of appropriate input and output indicators is a crucial prerequisite for using the DEA model to assess the operational efficiency of URRBMI and UEBMI. In establishing the evaluation framework, this study has summarized existing research on the use of the DEA model to assess social insurance efficiency. Notably, in the context of China, studies related to the operational efficiency of social insurance commonly employ fund input, the number of healthcare personnel, and the number of insured individuals as input indicators, while fund expenditure and indicators reflecting patient hospitalization characteristics are used as output indicators (Table 1).

**Table 1:** Summary of the input and output indicators about the health insurance efficiency

Author	Research object	Period of study	Methods	Input indicators	Output indicators
Liu et al. (2022) [23]	URRBMI	2017–2020	Three-stage DEA	Fund income; Number of health technicians; Government health expenditure	Fund expenditure; Epidemic morbidity rate; Resident health insurance expenditure
Li et al. (2023) [24]	UEBEI	2016–2020	Three-stage DEA	Fund income; Number of insured persons	Fund expenditure; Fund accumulated balance
Palazzolo (2018) [25]	Medicare ACOs	2013–2014	Two-stage DEA	Total expenditures per capita; Number of health technicians;	Beneficiaries Indicators; Earned Savings
Wu et al. (2015) [26]	Medicare	2003–2011	DEA-Tobit	Number of health technical employees; Quantity of beds in medical	Bed occupancy rate; Hospital physicians’ daily clinic visits; physicians’ daily hospital visits

Note: URRBMI, Urban and Rural Residents’ Basic Medical Insurance; UEBEI, Urban Employee’s Basic Endowment Insurance; ACOs, Accountable Care Organizations.

Input variables refer to the resources or factors utilized in the production process, representing the critical resources consumed by decision units in the production. On the other hand, output variables signify the outcomes or products generated by decision units through the utilization of these input variables. In developing the indicator framework, this study considered the principles of representativeness, objectivity, and feasibility. Combining these principles with expert opinions, the study ultimately determined the input indicators for URRBMI to be: fund income, the number of healthcare personnel per 10,000 people, and the number of insured individuals. For UEBMI, the input indicators include: fund income, the number of healthcare personnel per 10,000 people, the number of insured individuals, and the average wage of urban employees. The output indicators for both types of basic medical insurance were determined to be fund expenditure and hospitalization rate. Additionally, factors such as per capita inpatient costs, per capita outpatient costs, per capita fund balance, per capita cumulative fund balance, the actual reimbursement ratio for inpatients, per capita GDP, the number of medical institutions per 10,000 people, and the proportion of tertiary-level medical institutions were considered as variables influencing the comprehensive technical efficiency of the fund. Table 2 provides a detailed explanation of the indicators.

**Table 2:** Model variables

Category	Variable	Definition	Measurement
Input	Fund income	It is derived from the sum of funds contributed by various stakeholders and is primarily utilized to cover health insurance benefits and medical services	RMB 100 million yuan
	Number of insured individuals	It includes individuals enrolled in the basic medical insurance programs for urban or rural residents and urban employees	10,000 persons
	Number of health technical personnel	It comprises authorized doctors, registered nurses, pharmacists, laboratory physicians, radiologists, and other healthcare professionals	10,000 Persons
	Average salary of urban employees	The mean or average amount of salary earned by individuals employed in urban areas is referred to as the " average salary of urban employees “	RMB 1 Yuan
Output	Fund expenditure	It is used to cover a portion of the expenses for insured individuals during a medical procedure	RMB 100 million yuan
	Hospitalization rate (%)	The proportion of hospitalized individuals among the population covered by basic medical insurance	
Influencing factors	Per capita hospitalization cost	Average medical expenses incurred by hospitalized patients in each pooling area	RMB 1 Yuan
	Per capita outpatient cost	Average medical expenses generated by outpatient patients in each pooling area	RMB 1 Yuan
	Ln per capita GDP	It is referred to as the “logarithm of average per capita economic output	RMB 1 Yuan
	Per capita fund balance	It is the net difference between the annual income and expenditure of the residents’ basic medical insurance fund, divided by the year-end population	Yuan
	Per capita cumulative fund balance	It is the average accumulated surplus of funds per person	Yuan
	Actual reimbursement ratio	The percentage of medical expenses that can be received by a participant under the health insurance policy during a hospital stay	%
	Medical institution density	It represents the concentration or quantity of healthcare facilities within a specific area or population	10,000 persons
	Tertiary Hospital proportion	The percentage of tertiary hospitals out of the total number of public hospitals is represented by this metric	%

### Statistical test

#### Data envelopment analysis

Data Envelopment Analysis (DEA) was a widely used non-parametric technique for determining each decision-making units (DMUs) relative efficiency score and evaluating the linkages between inputs and outputs within different DMUs [27]. A linear programming model was employed to determine the weights assigned to each DMU, either minimizing inputs or maximizing yield, to gauge the relative efficiency levels concerning resource utilization and output. An advantage of DEA was its ability to manage the complexities associated with multiple inputs and outputs. By comparing the relative efficiency scores of DMUs, one could identify the most efficient DMU, with a score of 1 denoting optimal efficiency. The input-oriented BCC model (Variable Returns to Scale) was used in this study to evaluate efficiency. The linear form of the BCC model is as follows

1 $$ $$\begin{aligned} \begin{array}{c}Maximize\sum\limits _{r=1}^{s}{\mu }_{r}{y}_{r0}-{u}_{0},\\ subjectto\sum\limits _{i=1}^{m}{\omega }_{i}{x}_{ij}=1, \end{array} \\ \sum _{r1}^{s}{\mu }_{r}{y}_{rj}-\sum _{i=1}^{m}{\omega }_{i}{x}_{ij}-{u}_{0}\le 0, j=1,\dots n, \\ {\mu }_{r}\omega \ge 0, r=1,\dots S,i=1,\dots m.\end{aligned}$$

#### Malmquist index

The Malmquist index was a significant indicator used to measure changes in technical efficiency between two periods (usually involving two points in time) [28]. Its calculation relies on the DEA principle, where the Total Factor Productivity Efficiency (TFPCH) index was determined using a distance function. Efficiency Change (EFFCH) and Technical Change (TECHCH) are the two components of the Malmquist index, respectively, and Pure Efficiency Change (PHCH) and Scale Efficiency Change (SECH) are the two further components of EFFCH. The formula appears as follows:

2 $$ \begin{array}{c}{M}_{0}\left({x}_{m+1},{y}_{m+1},{x}_{m},{y}_{m}\right)={\left[\frac{{D}_{0}^{m}({x}_{m+1},{y}_{m+1)}}{{D}_{0}^{m}\left({x}_{m},{y}_{m}\right)}*\frac{{D}_{0}^{m+1}\left({x}_{m+1},{y}_{m+1}\right)}{{D}_{0}^{m+1}\left({x}_{m},{y}_{m}\right)}\right]}^{1/2}\end{array}$$

3 $$ \begin{array}{c}PECH\times SECH\times TECHCH=EFFCH\times TECHCH=TFPCH \end{array}$$

M_0_ represented the Total Factor Productivity Efficiency (TFPCH) index for the period (m + 1) relative to the period (m). Assuming the variable returns to scale, if M_0_ was more than 1, it indicates an improvement in production efficiency for that period; if M_0_ was less than 1, it signified a decrease in production efficiency.

#### Tobit model

Since the operational efficiency of URRBMI and URBMI fell within the range of 0 to 1, categorizing them as bounded dependent variables, this study employed the Tobit model to investigate the factors that influenced the efficiency of basic medical insurance operations in Gansu Province. The Tobit model, initially proposed by James Tobin, was widely utilized for handling truncated data [29]. Both the fixed effects panel data Tobit model and the random effects panel data Tobit model were simultaneously utilized in this study. The Stata program developed by Honor, Bo E. (1992) was employed for fixed effects panel data Tobit model fitting [30]. The Hausman test was used to choose between the panel data Tobit fixed effects model or the random effects model. Model fitting was conducted using Stata 15.0 software, with a significance level set at α = 0.05.

## Results

### Current status of the operation of the basic medical insurance

This study analyzed the operation of URRBMI and UEBMI in Gansu Province through non-parametric tests. Table 3 revealed significant differences between these two types in several aspects. Specifically, these differences were observed in fund income (*Z* = -2.89, *P* = 0.004), the number of insured individuals (*Z* = -6.566, *P* = 0.000), hospitalization rate (*Z* = -3.525, *P* = 0.000), fund expenditure (*Z* = -3.306, *P* = 0.001), cumulative fund balance (*Z* = -2.774, *P* = 0.006), and the actual reimbursement ratio for hospitalized patients (*Z* = -7.694, *P* = 0.000).

**Table 3:** Comparative analysis of the operation of the basic medical benefits fund

Basic health insurance fund operational indicators	Median (Quartiles)		N	Z	P
	Urban and rural resident	Urban employee			
Fund income	14.60 (13.55) ^*^	7.75 (4.4)	42	-2.89	0.004
Number of insured individuals	165.70 (143.45)	16.29 (5.40)	42	-6.566	0.000
Hospitalization rate	0.17 (0.05)	0.1899 (0.06)	42	-3.525	0.000
Fund expenditure	11.35 (10.93)	6.2 (3.20)	42	-3.306	0.001
Cumulative fund balance	6.27 (8.66)	10.9 (6.15)	42	-2.774	0.006
Actual reimbursement ratio	0.51 (0.07)	0.66335 (0.05)	42	-7.694	0.0

*Note* *Numbers in parentheses are Quartiles.

### DEA modeling results

As indicated in Table 4, from 2020 to 2022, the average TE of URRBMI operation in Gansu Province was 0.941, with an average PTE of 0.955 and SE of 0.985. In 2022, the TE was 0.957, showing a generally stable and slightly increasing trend, with 7 out of 14 (50%) DMUs operating on the efficiency frontier. Meanwhile, for UEBMI operation during the same period, the average TE was 0.900, with an average PTE of 0.923 and an average SE of 0.974. The overall trend exhibited an initial decrease followed by an increase. In 2022, the TE was 0.904, with 35.71% of the regions operating on the efficiency frontier.

**Table 4:** The efficiency of basic medical insurance at the municipal level in Gansu Province from 2020 to 2022 was evaluated using the input-oriented Banker, Charnes, and Cooper (BCC) model

Type of medical insurance	Year	PTE		SE		TE	
		Mean	Number of Efficient City	Mean	Number of Efficient City	Mean	Number of Efficient City
Urban and rural resident	2020	0.961	7(50.00%^*^)	0.977	5(35.71%)	0.939	5(35.71%)
	2021	0.947	7(50.00%)	0.990	7(50.00%)	0.938	7(50.00%)
	2022	0.957	7(50.00%)	0.989	8(57.14%)	0.947	7(50.00%)
Urban employee	2020	0.966	10(71.43%)	0.994	10(71.43%)	0.961	8(57.14%)
	2021	0.877	6(42.86%)	0.950	4(28.57%)	0.835	3(21.43%)
	2022	0.925	7(50.00%)	0.977	5(35.71%)	0.904	5(35.71%)

*Note* TE for total efficiency. PTE for pure technical efficiency. SE for scale efficiency.

^*^ Numbers in parentheses are the share of efficiency frontier regions.

### Results of the Malmquist index model

#### Malmquist index-URRBMI

**Fig. 2:**
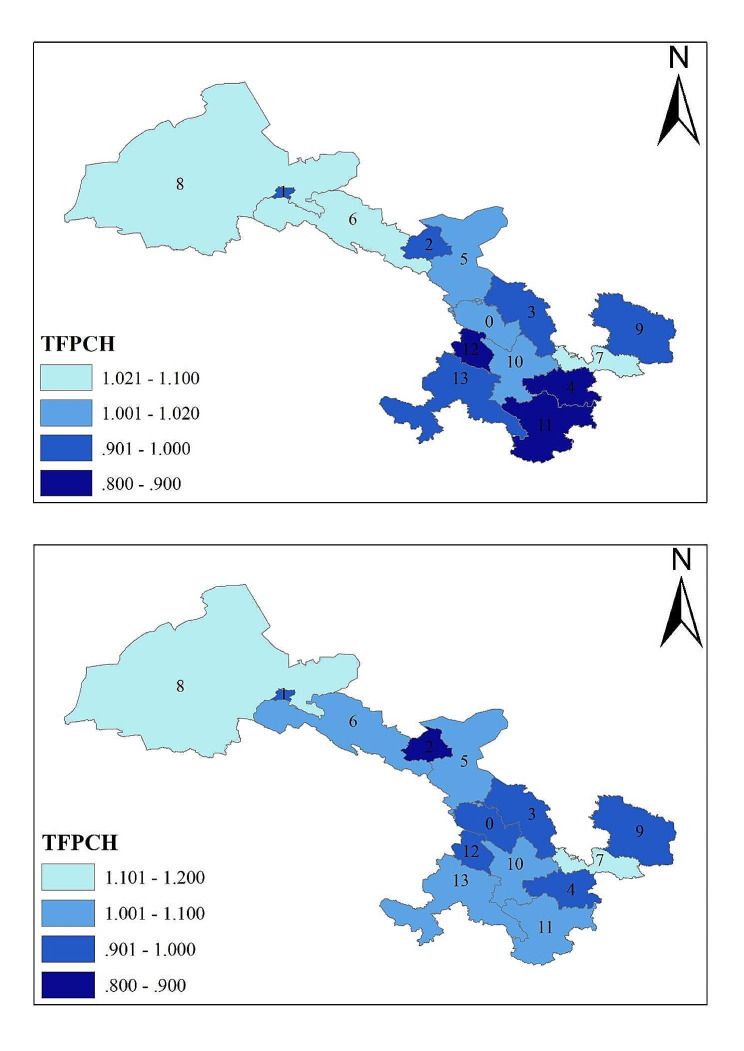
The variation in the total factor productivity changes (TFPCH) of the basic medical insurance for urban-rural residents in 14 municipalities during the period from 2020 to 2022

Figure 2 illustrates the regional distribution of the TFPCH index for URRBMI between 2020 and 2022. Over the study period, TFPCH indexes greater than 1.000 were observed in 6 cities (42.86%), indicating an increasing trend in the operational efficiency of URRBMI in these areas. Conversely, eight municipalities and prefectures (57.14%) had TFPCH indexes of less than 1.000, suggesting a declining trend in URRBMI’s operational efficiency in those regions. It is noteworthy that Jiuquan City (8), Zhangye City (6), and Pingliang City (7) had relatively higher TFPCH values, while Linxia Hui Autonomous Prefecture (12), Tianshui City (4), and Longnan City (11) exhibited relatively lower TFPCH values.

Table 5 presents the Malmquist indices for the operation of URRBMI in each city and prefecture. The TFPCH index for URRBMI from 2020 to 2021 was 1.036, with an EFFCH index of 0.998 and a TECHCH index of 1.039. From 2021 to 2022, the TFPCH index was 0.884, with an EFFCH index of 1.010 and a TECHCH index of 0.875. Over the period from 2020 to 2022, the average TFPCH was 0.957, indicating an overall declining trend in production efficiency. During the study period, the TECHCH was 0.953, contributing to the TFPCH being less than 1.000.

**Table 5:** Malmquist index-urban and rural resident basic medical insurance

Time	EFFCH	TECHCH	PECH	SECH	TFPCH
2020–2021	0.998	1.039	0.984	1.014	1.036
2021–2022	1.010	0.875	1.012	0.999	0.884
Mean	1.004	0.953	0.998	1.006	0.957

*Note* EFFCH for efficiency change, TECHCH for technical change, PECH for pure efficiency change, SECH for scale efficiency change, TFPCH for Total Factor Productive.

#### Malmquist index-UEBMI

**Fig. 3:**
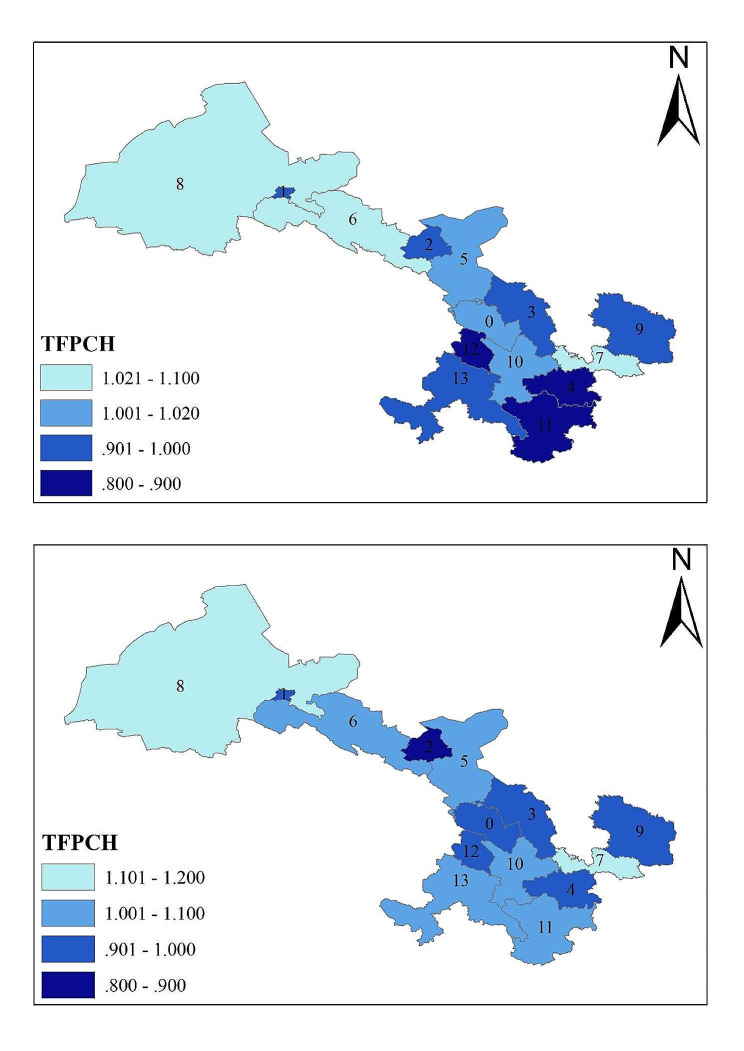
The variation in the total factor productivity changes of the urban employees’ basic medical insurance funds for in 14 municipalities during the period from 2020 to 2022

Figure 3 shows the regional distribution of TFPCH of UEBMI operation from 2020 to 2022. During this period, seven municipalities and prefectures (50.00%) had TFPCH indices of more than 1.000, indicating an increasing trend in the operational efficiency of UEBMI in these areas. Conversely, seven other municipalities and prefectures (50.00% of the total) had TFPCH indices of less than 1.000, suggesting a declining trend in UEBMI’s operational efficiency in those regions. It is noteworthy that Jiuquan City (8) and Pingliang City (7) had relatively higher TFPCH indices, while Jinchang City (2) had the relatively lowest TFPCH index.

Table 6 displays the Malmquist indices for UEBMI operation in various cities and prefectures. The TFPCH index from 2020 to 2021 was 1.035, exceeding 1.000, indicating an increase in the production efficiency of UEBMI operations in Gansu Province during this period. However, from 2021 to 2022, the TFPCH index was 0.957, below 1.000, suggesting a decrease in the production efficiency of UEBMI operations in Gansu Province during this period. When considering the data from 2020 to 2022, the TFPCH index was 0.995, also below 1.000, indicating a declining trend in the overall production efficiency of UEBMI operation in Gansu Province. The decrease in UEBMI’s TFPCH index owed to a reduction in the EFFCH index.

**Table 6:** Malmquist index-urban employees’ basic medical insurance

Time	EFFCH	TECHCH	PECH	SECH	TFPCH
2020–2021	0.858	1.206	0.900	0.954	1.035
2021–2022	1.091	0.878	1.060	1.029	0.957
Mean	0.968	1.029	0.977	0.991	0.995

*Note* EFFCH for efficiency change, TECHCH for technical change, PECH for pure efficiency change, SECH for scale efficiency change, TFPCH for Total Factor Productive.

### Panel Tobit model regression results

This study utilized the TE scores from the DEA models for the operation of basic medical insurance in various cities and prefectures from 2020 to 2022 as the dependent variable. Two separate models, the random effects panel data Tobit model and the fixed effects panel data Tobit model, were fitted, and the model selection using the Hausman Test. Table 7 indicated that the results of the random effects panel data Tobit model and the fixed effects panel data Tobit model were relatively consistent for the operational efficiency of the URRBMI. The Hausman Test results demonstrated that the fixed effects panel data Tobit model outperformed the random effects panel data Tobit model (*χ*^2^ = -2.32). However, for the operational efficiency of the UEBMI, there were significant differences between the results of the random effects panel data Tobit model and the fixed effects panel data Tobit model. The Hausman Test results revealed that the fixed effects panel data Tobit model was superior to the random effects panel data Tobit model (*χ*^2^ = -10.02).

**Table 7:** Comparison of Tobit panel data regression results on the operating efficiency of basic medical insurance funds (*N* = 42)^a^

Variable	Urban and rural resident		Urban employee	
	RE	FE	RE	FE
Per capita hospitalization cost	-0.0000574***	-0.0000515*	0.00000629	-0.00000574
	(-3.35)^b^	(-2.13)	(0.28)	(-0.13)
Per capita outpatient cost	0.00139**	0.00114	-0.00157	0.0000742
	(2.62)	(1.63)	(-1.60)	(0.06)
Per capita fund balance	-0.000132***	-0.000116***	-0.000106***	-0.0000787***
	(-3.76)	(-3.49)	(-4.15)	(-3.91)
Per capita cumulative fund balance	0.0000326*	0.0000363*	0.0000204**	0.0000014
	(2.45)	(2.05)	(2.64)	(0.09)
Ln per capita gdp	0.0568	0.0152	0.244***	0.225
	(1.54)	(0.13)	(3.98)	(1.15)
Tertiary hospital proportion	0.038	0.00674	0.00116	0.000247
	(0.69)	(1.20)	(0.04)	(0.03)
Actual reimbursement ratio	0.0042	0.00596*	0.0101	-0.0015
	(1.22)	(2.14)	(1.94)	(-0.42)
Medical institution density	0.000512	0.000217	-0.00179	-0.00314***
	(0.70)	(0.48)	(-1.73)	(-3.48)
Hausman test^c^	*χ*^*2*^ =-2.32		*χ*^*2*^ =-10.02	

*Note* *P < 0.05, **P < 0.01, ***P < 0.001.

RE represents the random effects panel data Tobit model, while FE represents the fixed effects panel data Tobit model.

^a^ Given the limited sample size and the collinearity among variables the study did not employ a mixed-effects model.

^b^Numbers in parentheses are test statistic Z-values.

^c^The Hausman Test examines whether to choose the FE model when *P* < 0.05 or the RE model when *P* > 0.05. The negative value of the Hausman test statistic indicates that the null hypothesis is untenable, and therefore, the FE model should be employed [31].

Table 8 presents the results of the fixed effects Tobit regression model for the efficiency of URRBMI operation. The results indicate that the lower the per capita hospitalization cost, the higher the operational efficiency (*β* = -0.000052, 95% *CI*: -0.000099 ∼ -0.000004, *P* = 0.033); the lower the per capita fund balance, the higher the operational efficiency (*β* = -0.000116, 95% *CI*: -0.000180 ∼ -0.000051, *P* = 0.000); the more per capita cumulative fund balance, the higher the operational efficiency (*β* = 0.000036, 95% *CI*: 0.000002 ∼ 0.000071, *P* = 0.041); and the higher the actual reimbursement ratio for hospitalized patients, the higher the operational efficiency (*β* = 0.005960, 95% *CI*: 0.000494 ∼ 0.011427, *P* = 0.033).

**Table 8:** Operating efficiency of urban and rural residents’ basic medical insurance fund: fixed effects panel data Tobit model regression results

Variable	Coefficient	SE	Z	P	95%CI	
Per capita hospitalization cost	-0.000052	0.000024	-2.13	0.033	-0.000099	-0.000004
Per capita outpatient cost	0.001136	0.000699	1.63	0.104	-0.000233	0.002506
Ln per capita GDP	0.015168	0.121114	0.13	0.900	-0.222211	0.2525469
Tertiary hospital proportion	0.006737	0.005632	1.20	0.232	-0.004302	0.017776
Per capita fund balance	-0.000116	0.000033	-3.49	0.000	-0.000180	-0.000051
Per capita cumulative fund balance	0.000036	0.000018	2.05	0.041	0.000002	0.000071
Actual reimbursement ratio	0.005960	0.002789	2.14	0.033	0.000494	0.011427
Medical institution density	0.000217	0.000449	0.48	0.629	-0.000663	0.0010973

Table 9 presents the results of the fixed effects Tobit regression model for the efficiency of UEBMI operation. The research findings indicate that the lower the per capita fund balance, the higher the operational efficiency (*β* = -0.0000787, 95%*CI*: -0.0001181 ∼ -0.0000392, *P* = 0.000); and the lower the medical institution density, the higher the operational efficiency (*β* = -0.0031376, 95%*CI*: -0.0049025 ∼ -0.0013726, *P* = 0.000).

**Table 9:** Operating efficiency of urban employees’ basic medical insurance fund: fixed effects panel data Tobit model regression results

Variable	Coefficient	SE	Z	P	95%CI	
Per capita hospitalization cost	-0.00000574	0.0000438	-0.13	0.896	-0.0000917	0.0000802
Per capita outpatient cost	0.0000742	0.0011944	0.06	0.950	-0.0022669	0.0024152
Ln per capita GDP	0.2245725	0.1958204	1.15	0.251	-0.1592284	0.6083734
Tertiary hospital proportion	0.0002468	0.0088149	0.03	0.978	-0.0170302	0.0175238
Per capita fund balance	-0.0000787	0.0000201	-3.91	0.000	-0.0001181	-0.0000392
Per capita cumulative fund balance	0.0000014	0.0000161	0.09	0.931	-0.0000301	0.0000329
Actual reimbursement ratio	-0.0014979	0.0035952	-0.42	0.677	-0.0085443	0.0055485
Medical institution density	-0.0031376	0.0009005	-3.48	0.000	-0.0049025	-0.0013726

## Discussion

This study employed the DEA-Malmquist-Tobit model to assess the operational efficiency of basic medical insurance in the Gansu region of China and conducted an in-depth exploration of its influencing factors. The results revealed that the average TE score for URRBMI was 0.941, showing a generally stable trend with slight improvement, while UEBMI had an average TE score of 0.900 with noticeable fluctuations. From 2020 to 2022, the TFPCH for URRBMI was 0.957. The decrease in TECHCH explained the decline in overall production efficiency during 2021–2022. For UEBMI, the TFPCH was 0.995, with EFFCH being less than 1.000, serving as a primary reason for TFPCH being below 1.000. The study found that a rise in per capita hospitalization expenses and per capita fund balance negatively affected the operational efficiency of URRBMI. Conversely, an increase in per capita cumulative fund balance and the actual reimbursement ratio had a positive impact. For UEBMI, its operational efficiency decreased with an increase in per capita fund balance and medical institution density.

From 2020 to 2022, the average TE of URRBMI in Gansu Province was 0.941, while that of UEBMI was 0.900. China’s URRBMI from 2017 to 2020 was estimated to have a TE of 0.921 compared to other research using comparable methodologies [23]. Some research has characterized Gansu Province’s URRBMI as a “low-input, high-efficiency” fund operation model [32]. The average TE of China’s UEBMI from 2017 to 2019 was 0.817 [33]. Additionally, studies have affirmed that regions with lower economic levels can more effectively utilize healthcare resources [34–36]. Throughout the study period, URRBMI outperformed UEBMI in terms of both average TE and stability. The outbreak of COVID-19 at the end of 2019 posed significant threats to global economic development, social stability, and human health, particularly challenging healthcare systems. Gansu Province experienced two waves of pandemic peaks in 2021 and 2022. In Gansu Province, URRBMI had implemented city-level pooling earlier than UEBMI. Relevant studies have reported that enhancing the pooling level of healthcare insurance can improve its operational efficiency and risk resilience [11, 37]. That could be one of the reasons why the average level and stability of URRBMI’s operational efficiency during the pandemic period were superior to those of UEBMI.

The results of the Malmquist index analysis indicate that the average TFPCH index for both URRBMI and UEBMI from 2021 to 2022 was below 1.000, suggesting an overall decline in the efficiency of healthcare resource utilization within the basic medical insurance systems during this period. In the case of URRBMI, the TFPCH below 1.000 was driven by TECHCH. On one hand, due to the COVID-19 pandemic, a significant portion of healthcare resources was allocated to managing the outbreak and researching COVID-19 treatments [38], resulting in a temporary reduction in the supply of certain healthcare services. On the other hand, to mitigate the risk of infection, some patients postponed or avoided non-urgent medical assistance, leading to a temporary decrease in the demand for healthcare services [39, 40]. While this enhanced financial stability for the fund management agencies of URRBMI, it may have translated to reduced accessibility to healthcare services for insured individuals. However, for UEBMI, the TFPCH below 1.000 was attributed to EFFCH. Suggests that UEBMI may face multifaceted challenges. Firstly, the healthcare system experienced significant stress due to the impact of COVID-19 [38]. Secondly, in 2022, Gansu Province’s UEBMI had just achieved municipal-level pooling, and the department responsible for the fund might not yet be familiar with the policies and management methods. Indicates that the fund management agencies for UEBMI need to address issues related to inadequate resource utilization, improvements in fund operation strategies, and sustainability. For insured individuals, these challenges may impact the quality of services and hinder the satisfaction of healthcare needs.

Per capita hospitalization expenses show a negative correlation with the operational efficiency of URRBMI. First off, as per capita hospitalization costs increase, the health insurance fund is put under more financial strain, which could result in diminished solvency or even financial difficulties. Second, the irrational distribution of healthcare resources among districts may be exacerbated as costs increase, thus reducing the efficiency of the fund and affecting the provision of other healthcare services [41].

Per capita fund surplus exhibits a negative correlation with the operational efficiency of URRBMI and UEBMI, whereas per capita accumulated fund surplus shows a positive correlation with URRBMI. The outbreak and widespread prevalence of COVID-19 at the end of 2019 led to a decrease in the accessibility of healthcare services [42, 43], resulting in the underutilization of essential medical insurance funds, leading to resource wastage and diminished efficiency. Maintaining a moderate level of accumulated fund surplus contributes to enhancing the financial robustness of the fund, ensuring its stability when faced with sudden increases in healthcare expenses or other emergencies. In turn, this supports the sustainability of the fund. Furthermore, the rise in accumulated fund surplus also provides greater flexibility for healthcare insurance, such as expanding coverage or improving service quality, thereby enhancing the fund’s efficiency and service levels and benefiting a larger population.

Per capita fund balance is negatively correlated with the operational efficiency of URRBMI and UEBMI. The outbreak and widespread prevalence of COVID-19 at the end of 2019 led to a decrease in the accessibility of healthcare services [42, 43], resulting in the underutilization of basic medical insurance funds, leading to resource wastage and diminished efficiency. However, per capita cumulative fund balance exhibits a positive correlation with URRBMI. Maintaining a moderate level of accumulated fund surplus contributes to enhancing the financial robustness of the fund, ensuring its stability when faced with sudden increases in healthcare expenses or other emergencies. In turn, supports the sustainability of the fund. Furthermore, the increase in accumulated fund surplus also provides greater flexibility for healthcare insurance, such as expanding coverage or improving service quality, thereby enhancing the fund’s efficiency and service levels, benefiting a larger population.

The UEBMI fund’s operational efficiency decreases as healthcare facility density rises. This tendency may be explained by the fact that beneficiaries of the UEBMI receive higher actual reimbursement rates for inpatient care than those whose insurance is provided by the URRBMI. As a result, participants in the UEBMI program may experience problems with excessive use of medical services, which wastes medical resources and makes insurance administration more difficult. In turn, the UEBMI’s operational effectiveness suffers as a result.

Against the backdrop of the COVID-19 outbreak and the proactive efforts of the Gansu Provincial Medical Insurance Bureau to promote the level of pooling of basic medical insurance, this study used the DEA-Malmquist-Tobit model to objectively and scientifically evaluate the operational efficiency. The findings of this study hold value for policymakers and healthcare insurance management agencies in formulating and improving policies. However, this study does have certain limitations. Firstly, the Gansu Provincial Medical Insurance Bureau was established in November 2018. Consequently, this study analyzed data on the operation of the basic medical insurance fund only for the years 2020 to 2022. The relatively short period may impact the long-term trends and stability of the research findings. Future studies may benefit from considering a broader time range. Secondly, this research predominantly focused on the Gansu region of China, potentially limiting the generalizability of the results. This limitation restricts the ability to make broad inferences to other areas. Therefore, future research could explore comparisons between multiple regions to gain a more comprehensive understanding of the variations and commonalities in the operational efficiency of healthcare insurance funds. In summary, despite these limitations, this study provides valuable insights into the operational efficiency of the medical insurance fund in Gansu Province. It serves as a beneficial reference for future research and policy formulation.

## Conclusions and policy recommendations

### Conclusions

In this study, we conducted a comprehensive analysis of the operational efficiency of the basic medical insurance system in Gansu Province over the past three years using the DEA model, Malmquist total factor productivity index, and Tobit model. Fund revenue, the number of healthcare professionals per 10,000 population, and the number of insured individuals were considered as input indicators. Fund expenditure and hospitalization rate were taken as output indicators. The main findings of the study are summarized below:

1. By comparing the operational efficiency of URRBMI and UEBMI, as well as the efficiency of UEBMI before and after the enhancement of the pooling level, this study revealed that URRBMI outperforms UEBMI in terms of average efficiency and stability in fund operation. It is noteworthy that the operational efficiency of UEBMI experienced a significant decline from 2020 to 2021. However, after the implementation of municipal-level pooling at the end of 2021, its efficiency showed improvement. Therefore, the study concludes that enhancing the pooling level can effectively improve the operational efficiency and risk resistance of the basic medical insurance system.
2. From the perspective of financial management of medical insurance funds, an analysis of the influencing factors of URRBMI and UEBMI revealed a negative correlation between per capita fund balance and the operational efficiency of both systems. However, the per capita cumulative fund balance is positively correlated with the operational efficiency of URRBMI. Consequently, the study concludes that financial management of medical insurance funds is a crucial factor influencing the operational efficiency of basic medical insurance.
3. Regarding the incentive mechanism of basic medical insurance, the analysis in this study indicates a positive correlation between the operational efficiency of URRBMI and the actual reimbursement ratio for hospitalized patients. Thus, the study suggests that the operational efficiency of URRBMI is significantly influenced by the incentive mechanism of basic medical insurance.
4. From the perspective of healthcare service costs, the study found a negative correlation between per capita hospitalization costs and the operational efficiency of URRBMI. Consequently, the study concludes that high healthcare service costs will lead to a decrease in the efficiency of URRBMI.

### Policy recommendations

Based on the conclusions drawn from our study, the following recommendations are proposed:

**(1) Control the Growth of Per Capita Hospitalization Costs**:

The increase in per capita hospitalization costs has adversely impacted the operational efficiency of the fund. Therefore, it is recommended that healthcare insurance management authorities strengthen their monitoring of healthcare service prices and quality, while actively promoting the implementation of tiered diagnosis and treatment policies. This will contribute to curtailing the unnecessary escalation of per capita hospitalization costs, thereby enhancing the efficiency of the fund.

**(2) Strengthen Financial Management of the Fund**:

The rise in per capita fund surplus has had unfavorable effects on fund efficiency. Hence, close attention to the financial status of the fund is warranted. Healthcare insurance management authorities may consider formulating rational fund utilization policies to ensure funds are allocated to meet the medical needs of the insured while maintaining an appropriate fund surplus for unforeseen circumstances. This approach will ensure the judicious use of funds and long-term sustainability.

**(3) Optimize the Basic Medical Insurance Incentive Mechanism**:

The positive impact of an increased actual reimbursement ratio on fund operational efficiency is noted. Therefore, it is suggested to implement tiered diagnosis and treatment policies and judiciously raise the actual reimbursement ratio for hospitalized patients within reasonable limits. This measure will incentivize healthcare service providers to deliver more efficient and economical medical services, contributing to the optimization of the basic medical insurance operational framework.

**(4) Elevate Coordination Levels Further Upon Improving Municipal-Level Integration of Basic Medical Insurance**:

Our study reveals that elevating coordination levels significantly benefits the operational efficiency and risk resilience of the basic medical insurance fund. Consequently, it is recommended that Gansu Province, building upon improvements in municipal-level integration, actively advances provincial-level integration of basic medical insurance to further enhance the overall efficiency of the insurance system. This proactive step will better equip the system to cope with risks and elevate the overall effectiveness of the medical insurance system.

## Data Availability

The dataset used during the current study is available from the corresponding author on reasonable request.

## Declarations

### Author information

Institute of Epidemiology and Health Statistics, School of Public Health, Lanzhou University, Lanzhou, Gansu, China.

Feng Hu, Liu Heming, Cao Wenxuan, Wang Xuemei, Hu Xiaobin.

Gansu Provincial Medical Insurance Service Centre, Gansu, China.

Liang Qijun.

## Abbreviations

DEA: Data Envelopment Analysis
URRBMI: Urban and rural residents’ basic medical insurance
UEBMI: Urban employees’ basic medical insurance
DMUs: Decision-making units
TE: total efficiency
PTE: pure technical efficiency
SE: scale efficiency
EFFCH: Efficiency change
TECHCH: Technical change
PECH: Pure efficiency change
SECH: Scale efficiency change
TFPCH: Total factor productive efficiency change
